# Sickle Cell anemia and hearing loss among children and youngsters: literature review

**DOI:** 10.1590/S1808-86942012000100020

**Published:** 2015-10-20

**Authors:** Luzia Poliana Anjos da Silva, Camila Vila Nova, Rita Lucena

**Affiliations:** a MSc and PhD in Medicine and Health by the Graduate Program in Medicine and Health – Federal University of Bahia (UFBA); Adjunct Professor of the Health Sciences Institute of Health (ICS-UFBA). Specialist in Audiology, Neonatology and Collective Health.; b PhD in Medicine and Health – Graduate Program in Medicine and Health -UFBA. Professor at UNIME and UNIJORGE (Bahia); c Neuropediatrician; PhD in Neurosciences - UFBA – Adjunct Professor in Neurology - FAMEB - UFBA, Adjunct Professor of Neurology - FAMEB - UFBA. Graduate Program in Medicine and Health -PPGMs –UFBA. Doctorate Program in Medicine and Health.

**Keywords:** anemia, sickle cell, deafness, prognosis, public health

## Abstract

Sickle cell anemia is still a significant public health issue in underdeveloped and developing countries. Sickle cell disease is one of the most common inherited diseases in Brazil. It affects mainly the mixed race population. Approximately 1 African-Brazilian child is affected with sickle cell disease for every 37,400 children born alive. Hearing loss has been considered one of the main clinical manifestations, especially in children. However, to date, there are just a hand full of studies in Brazil and the Brazilian state of Bahia has the largest African-descended population, attempting to establish the frequency of this event.

**Objectives:**

To analyze the major studies associated with the subject, published in the last twenty years in the main indexed databases.

**Methods:**

To use MEDLINE to identify the main papers published in English in medical literature, between January of 1989 and January of 2009; associating sickle cell anemia and hearing loss, with its clinical outcomes.

**Conclusion:**

Given that it is always possible to attempt to prevent disabilities, understanding hearing loss in children with sickle cell anemia enables to maximize quality of life and provides for a broader school attendance.

## INTRODUCTION

Sickle cell (SC) anemia is a hereditary hemoglobin disorder associated with a very specific molecular lesion, which is the exchange of glutamic acid for valine in the 6th residue of the hemoglobin beta chain, originating the S hemoglobin^1^.

Sickle cell anemia is still a public health issue in underdeveloped and developing countries. The sickle cell disease includes a group of genetic alterations characterized by the predominance of the S hemoglobin, which causes red blood cells to have the shape of a sickle. The main clinical manifestations of sickle cell anemia are a chronic anemia caused by the destruction of red blood cells (hemolytic type) and thrombotic events. Despite a variable disease severity, individuals affected require regular health care from childhood all the way to adult age^2^.

The S hemoglobin gene is highly frequent throughout the Americas, and in Brazil it is more frequent in the southeast and the northeast^3^. The S hemoglobin (Hb**S**) reaches the frequency of 7.6% in the population from the Brazilian northeast, made up of 82% of blacks and browns^3^.

Hearing deficit is described as one of the symptoms which happen because of the cochlear high sensitivity to vessel occlusion, causing ischemia and cochlear anoxia, because of the sickle cells which preclude blood flow to the cochlear epithelium^4^. The fact that the cochlea is mainly fed by one single artery, the labyrinthine artery, which can be a terminal artery, makes the inner ear very much prone to circulatory changes^5^.

The concern with the early diagnosis of hearing impairment has been a constant issue, since the loss caused by such impairment, often times is irreversible, affecting not only oral language, but also the child's global development and school performance^6^.

Although sickle cell anemia has been broadly studied in Brazil in terms of population frequency and clinical manifestation, its public health issues associated with child-youth hearing loss has not received proper attention in our country.

In the present paper we reviewed the literature on sickle cell anemia and its clinical-audiologic repercussions in children and adolescents, analyzing how the specialized literature discusses the topic in different methodological approaches.

## OBJECTIVE

To use the literature from the past two decades to analyze which are the main findings on the dual: sickle cell anemia and hearing loss, and the later repercussions in individuals with sickle cell anemia.

## MATERIALS AND METHODS

### Identification and selection of papers

Using the MEDLINE database, we studied papers in the English language, published in the medical literature between January of 1989 and January of 2009, which reported associations between sickle cell anemia and hearing deficit, and its clinical repercussions. We reviewed Medline, Scielo, and Caribbean and Latin-American Literature on Health Sciences, with the following keywords: *sickle cell disease; hearing loss; auditory dysfunction; stroke; prevalence; audiological evaluations; auditory electrophysiological assessments,* used alone or in combination in our study. Based on the analysis of a list bearing one hundred and fifty-seven (157) references of relevant publications and review papers, we selected fifty-eight (58) papers which were the most relevant concerning the topic at hand.

## LITERATURE REVIEW

### Sickle Cell Anemia

Sickle cell anemia has an unknown origin, but it very likely started in Africa, millions of years ago. It protects people against malaria, a common and severe ailment of warm weather countries. The first case described in contemporary medicine literature happened in 1910, by J. Henrick, and since then, numerous associations between the disease conditions and its complications have been described^7^.

The disease started in Africa, and it was brought to the Americas by the forced immigration of slaves. In Brazil, it is heterogeneously distributed, being more frequent where there are higher numbers of African descendants (northeast). Besides Africa and the Americas, it is today found all over Europe and in large regions of Asia^8^.

In the USA, sickle cell anemia is considered a significant public health problem; one in every 400 African-Americans is treated for sickle cell anemia in the public health care system^9^.

Sickle cell anemia, as clinical expression of the homozygosis of the S hemoglobin gene, is an important genetic abnormality in Brazil, especially in the regions which received large numbers of African slaves. In our country it is estimated that 3 for every 500 Brazilian-Africans have sickle cell anemia trace and one for every 500 blacks in Brazil is born with some form of the disease. Although there is a higher prevalence in African-descendants, the Caucasian population, especially those from the Mediterranean (Greece, Italy, etc.), Middle East, India, have many cases of sickle cell disease^10^.

In our country, the disease was considered predominant among blacks and browns; nonetheless, new studies have shown that although the disease predominates among African-descendants, the growing mixture of races is opening the borders of this disease, unbundling it from ethnic links^11^.

In the state of Bahia, it was estimated that 5.5% of the general population bears a sickle cell trace, reaching 6.3% of the African-descended population^12^. One study carried out with African-descended children in the city of Salvador, pointed to an even higher frequency: 7.4%^13^.

The decrease in the levels of O_2_ causes morphological polymerization of the red blood cell, and it takes on an anomalous form, looking like a sickle. The elongated deformed red cells, not always can cross through small vessels, blocking them and preventing blood flow in the vicinity areas. The disease's course is variable; there are patients who have severe problems with greater frequency and others who have only sporadic health problems.

In general, besides chronic anemia, the different forms of sickle cell diseases are characterized by numerous complications which may affect almost all organs and systems, with relevant morbidity, reduction in work capacity and in life expectancy. Besides chronic anemia manifestations, the clinical picture is dominated by osteocartilaginous pain episodes, abdominal pain, pulmonary infarctions and infections, growth and sexual maturation delays, stroke and chronic involvement of multiple organs, systems or body aparatuses^14^.

Usually, it is during the second half of the first year of the life of a child that the first symptoms of the disease manifest, except for the cases in which the blood test – to detect the disease, was carried out at birth or when the baby is in the nursery. It is common for the disease to manifest itself by the time the child reaches school age, and it is rare when this does not happen.

As per institutional policy, sickle cell anemia started to be “appreciated” in 1996 through the Sickle Cell Anemia Program of the Ministry of Health (PAF-MS) which intends, within its general goals, to “promote educational actions, aiming at educating the population about the disease; to train health care professionals for prevention, diagnosis and treatment of the disease, and also to promote the active search for affected people”^15^.

### Sickle Cell Anemia and Hearing Deficit

Sickle cell anemia is a world-wide disease, especially present in countries with blacks and mixed populations, where the SS sickle cell form is very common. A reduction in O_2_ levels causes the morphological polymerization of the red cell and it takes on an anomalous form resembling a scythe or sickle. The morphological change associated with the physiological change in the structure of the red cells drastically reduces the capacity to transport O_2_ through the body. Some authors report that the hearing deficit concurrent to sickle cell anemia is not associated with the classic symptoms, but rather to its pathogenesis.^4^

The relationship between sickle cell anemia and hearing loss has been clearly documented. There are numerous investigations correlating the peripheral sensorial hearing deficit with a large variety of results. The prevalence of hearing deficit reported in the literature describes losses of mild to profound levels. Most of the cases have bilateral hearing loss; unilateral hearing loss is rarely described.^16^, ^17^, ^18^, ^19^

A metanalysis study carried out on the hearing of individuals with sickle cell reported that the ischemia caused to the stria vascularis leads to hypoxia in the organ of Corti, especially in individuals with a large number of painful spells, which would indicate a significant risk for the entire hearing system.^20^

Some studies have reported that the cochlear lesion is caused by the deformation of the red blood cell, which prevent proper blood supply to the high metabolic activity required to maintain the delicate ionic and electrical balance of the endolymph, and that the anoxia caused to the organ of Corti would cause extensive and progressive cochlear damage, which would justify the lack of otoacoustic emissions in individuals with sickle cell anemia.^21^

There are authors who have analyzed distortion product otoacoustic emissions (DPOAE) in two groups of twenty African-American children with and without sickle cell anemia, and concluded that the DPOAE were changed in children with sickle cell, especially in relation to an increase in DPOAE amplitude.^22^

A case-controlled study carried out in the USA, stratifying groups with different types of hemoglobin diseases (SS, SC, SB-thalassemia) for hearing assessment using Brainstem Auditory Evoked Potential (BAEP) to study the threshold, showed that the SS form had 80% of the individuals with a level of hearing impairment varying from mild to profound.^23^

Numerous studies (between 1988 and 2004) used different methodologies to determine the hearing impairment in individuals with sickle cell, from conventional audiologic evaluation (tonal and vocal audiograms), central auditory processing assessment, brainstem auditory evoked potential and otoacoustic emissions. The results obtained had different percentage values of hearing deficit incidences, varying in the population groups studied between 12 and 66% among individuals with sickle cell anemia. One of the most employed techniques was the study of otoacoustic emission amplitudes, which showed a considerable increase in the amplitude of otoacoustic emissions, bearing a considerable increase in response amplitude from children with sickle cell anemia.^24^, ^25^, ^26^, ^27^, ^28^

In African countries, such as Congo, with a predominance of black individuals, a study reported sudden hearing loss in individuals with sickle cell. The pathophysiology of the hearing loss was found to be associated with the fact that the vascular occlusion caused obliteration of the internal artery of the auditory terminal, thus causing ischemia and cochlear anoxia. The high sensitivity of the cochlea towards anoxia and its great fragility concerning external factors require an accurate and early audiological diagnosis, and also specialized care. The authors stress the need for regular audiological follow up in patients with sickle cell anemia.^4^

In Europe, there was a study carried out in Spain, with the Mediterranean population, describing two cases of sudden hearing loss in patients with sickle cell anemia, stressing the high cochlear sensitivity to arterial occlusion caused by the malformation of the red cells. The authors stress the need for prevention, diagnosis and treatment of the hearing loss caused by vascular occlusion led by sickle cell disease; and also the need for public health policies which support the rehabilitation of sickle-cell individuals with sensorineural hearing loss^29^.

A case-control study carried out in Nigeria, investigated the hearing function of 62 individuals at an age range between 7 and 30 years, with sickle cell anemia, found a sensorineural hearing loss above 30 dB in 40% of the individuals evaluated and in 5.5% in the control group. Hearing impairment installed progressively along the disease course, bilaterally and it reached speech frequencies. The authors stress the need for prevention, diagnosis and treatment of the hearing loss caused by the vascular occlusion which happens in sickle cell anemia, and also the need for public health policies which support the rehabilitation of sickle cell individuals with sensorineural hearing loss^30^.

In Ghana, they carried out a study to establish the prevalence of sensorineural hearing loss in patients with sickle cell disease. Twenty-nine percent of the patients with sickle cell anemia had moderate sensorineural hearing loss, especially in the frequency range between 4 and 8 KHz. In the study, the authors stress the need for studies with populations from different geographical areas for prevalence analysis.^31^

A case-control study carried out to assess hearing in patients with sickle cell anemia in Nigeria, showed that the hearing impairment reaches more the speech frequencies, and that the hearing deficit happens predominantly in the inner ear, having seen that tympanometric curves are normal, ruling out this type of problem in the middle ear (otitis), which could cause the hearing impairment^32^.

In Kenya, a retrospective study was carried out with 360 patients in the age range between 7 months and 21 years, with bearers of the SS form of the sickle cell disease, who were submitted to neurological assessment. Numerous neurological sequelae were found: 67% of the patients had already had a stroke; 33%, seizures, visual disorders and hearing impairment, besides cerebellar degeneration, mental confusion and hallucinations. Multiple neurologic complications were described in 4 patients. The authors ratify that in Kenyan patients, there is the need for longitudinal audiological and neurological follow up in individuals with sickle cell anemia.^33^During the literature review carried out for this study, we found only one paper from southern Brazil, reporting hearing deficit associated with sickle cell disease in adults. The authors reported the occurrence of sensorineural hearing loss in 21.4% from the sickle cell disease group, compared to 3.6% from the control group without sickle cell anemia. The study indicated that southern patients with sickle cell anemia had a predisposition do develop sensorineural hearing loss when compared to the general population.^34^

Considering the major racial mix in Bahia, with a predominance of the black population, it is extremely relevant to know about and react early on concerning hearing impairment, especially concerning the pediatric population, in order to install prevention and diagnostic measures early on so as to avoid learning deficits and late schooling.

According to estimates from the World Health Organization, every year in Brazil we have about 3,500 children born with sickle cell disease. Twenty percent of them will not reach five years of age, because of complications directly associated with the disease itself. Only early diagnosis and proper treatment can change this scenario. According to data from APAE-Salvador, from August of 2000, when the “foot test” started, neonatal screening for hemoglobin diseases, allowed us to know the true incidence of such disease: 1,655 live newborns with sickle cell anemia and 1 child with sickle cell trace for every 17 births^35^.

Based on the analysis of studies carried out to date, we can see that knowing the magnitude of sickle cell disease, with many analysis variables and sequelae, hearing impairment in children and adolescents, often times is ultimately missed or underdiagnosed, which impairs early specialized intervention, especially in children acquiring oral language or in those starting school.

Different diagnostic methods confirmed the great variety in the degree of hearing loss in patients with sickle cell disease; nonetheless, the sensorineural hearing loss type predominated in the studies considered, confirming the damage caused to the cochlea and the auditory nerve, caused by hypoxia stemming from the sickle shape of the red cells.

Thus, we emphasize the early detection of hearing loss through otoacoustic emission tests, which can be carried out together with the PKU test, contributing to a reduction in severity and facilitating early and systematized intervention.

The Sickle Cell Anemia Program, developed by the Department of Health, with a very active participation of African-Brazilian activists as of the late 90's, has tried to conceptualize that sickle cell anemia is much more than a disease, under to auspices of medicine alone^36^.

In the state of Bahia, in June of 2003, a program was installed for the prevention, diagnosis and integral medical care and education to people with sickle cell disease and other hemoglobin diseases, including the Brazilian Public Health system – SUS - in this program we can guarantee that it will cover the diagnostic tests for hemoglobin diseases for newborn children, as well as specific treatments for the sickle cell disease.

Therefore, as it is guaranteed by law, we believe it to be of fundamental importance to inform the families of these patients about their rights guaranteed by law which are often times unknown to them, and its clinical repercussions and sequelae so as to provide early and efficient treatment in order to minimize the repercussions it has on the linguistic and cognitive development of children with sickle cell anemia.

## CONCLUSIONS

Having said this all, based on the literature review carried out, it is clear the need to prevent, diagnose and systematically follow up these children with sickle cell anemia, because hearing loss, when underdiagnosed or diagnosed very later on, may cause irreparable damage to the sickle-cell-affected individual's linguistic, biopsychosocial and emotional developments.
